# No evidence for kin selection as an explanation for social group formation in clown anemonefish

**DOI:** 10.1093/beheco/araf075

**Published:** 2025-06-29

**Authors:** Theresa Rueger, Tina Adria Barbasch, Matishalin Patel, Steven Michael Bogdanowicz, Peter Michael Buston

**Affiliations:** Dove Marine Laboratory, School of Natural and Environmental Sciences, Newcastle University, King’s Gate, Newcastle upon Tyne, NE1 7RU, United Kingdom; Carl R. Woese Institute for Genomic Biology, University of Illinois Urbana Champaign, 1206 W Gregory Dr, Urbana, IL 61801, United States; Department of Biological and Marine Sciences and the Centre for Excellence in Data Science, AI, and Modelling, University of Hull, Cottingham Rd, Hull, HU6 7RX, United Kingdom; Department of Ecology and Evolutionary Biology, Cornell University, 215 Tower Rd, Ithaca, NY 14853, United States; Department of Biology and Marine Program, Boston University, Boston, 5 Cummingham Mall, MA 02215, United States

**Keywords:** social evolution, cooperative breeding, reproductive skew, relatedness, coral reef fish

## Abstract

Social groups in which some individuals forgo reproduction and others reproduce, are one of the most remarkable products of evolution. To fully understand these social groups, we must understand both why non-breeders tolerate their situation and why breeders tolerate non-breeders. In general, breeders tolerate non-breeders because they help provision the breeders’ offspring or the breeders themselves, but in some vertebrate societies the benefits that breeders accrue from non-breeders are surprisingly hard to detect. This raises the question: why do breeders tolerate non-breeders in such societies? Here, we test the hypothesis that breeders of the clown anemonefish (*Amphiprion percula*) will tolerate non-breeders because they are distant relatives who go on to inherit the territory. We use 40 polymorphic microsatellite loci to assess the pairwise relatedness of 683 individuals from 203 groups. We show that the mean pairwise relatedness among individuals from the same group is effectively zero, and no different from that found among individuals from the same reef or that found among individuals from the population at large. Further, we show that the mean pairwise relatedness found among breeder/breeder dyads is no different from that found among breeder/non-breeder dyads or that found among non-breeder/non-breeder dyads. We conclude that kin selection does not explain why breeders tolerate non-breeders in the clown anemonefish, and suggest that the explanation must lie with other, as yet untested, hypotheses: within-generation bet-hedging or mutualist-mediated benefits.

## Teaser

The evolution of social group living is often explained by close relatives sharing resources. However, it is unclear if this explanation works for marine fishes that live in social groups. We investigate this in the group-living clown anemonefish and found no indication of close relatives living together. In marine environments, alternative explanations must be considered, which may be an opportunity to enhance our understanding of the evolution of social groups in general.

## Introduction

The formation and maintenance of social groups where some individuals reproduce and others forego reproduction is largely, though not completely, explained through kin selection (Bourke 2011). Under the kin selection hypothesis individuals forego reproduction, perform costly helping behavior, or share resources because it benefits their close relatives’ offspring (Hamilton 1964). Kin selection can then explain why non-breeding individuals in social groups accept their fate. By helping their parents or siblings reproduce they ensure their genes are transmitted into the next generation (Woolfenden and Fitzpatrick 1984; Emlen and Wrege 1988; Clutton-Brock 2002; Griffin and West 2003). It can also explain why breeders tolerate non-breeders in their territory even if they do not provide direct help; the breeder gains inclusive fitness through a related non-breeder inheriting the breeding territory and eventually producing offspring (Kokko and Johnstone 1999).

While kin selection is the preeminent hypothesis explaining social group formation, social groups can form because of a combination of indirect and direct benefits and the structure and dynamics of groups is often explained by this interplay. For example, in the paper wasp *Polistes dominulus* there is a rigid queuing structure where groups of females (foundresses) find and build nest sites but one female (dominant) will suppress the reproduction of the other females (subordinate helpers) (Queller et al. 2000). However, the relatedness structure in this species is complicated, with high pairwise relatedness of foundresses during the breeding season, but much lower relatedness in over-wintering aggregations. This reflects the shift from indirect to direct benefits: During breeding the nest is raising a brood of the dominant, so indirect fitness is exploitable and high relatedness groups are formed, while over-wintering primarily affects survival and direct fitness benefits are the primary fitness effects, making group-relatedness less important. Even during the breeding season non-related females will still queue as nest sites are limited and direct fitness benefit of inheriting a nest site is large (Toyoizumi and Field 2014). In Pied Kingfishers, *Ceryle rudis*, there is a clear distinction between dominant tolerance of primary and secondary helpers (Reyer 1990). Primary helpers, mostly related males from previous broods that cannot find their own nest sites, are well tolerated and feed the dominants’ offspring and protect the nest at a similar level of investment as their parents. On the other hand, secondary helpers are unrelated males that show a reduced level of investment, are less well tolerated and only realize their investment when the dominant pair is broken and they can inherit the nest or if they access extra-pair mating opportunities.

These examples show that relatedness can be diagnostic when assessing whether the helpers in a social group help due to indirect fitness effects or direct fitness effects between the helper class and the dominant. They also show that helpers and dominants modify their behavior based on expected indirect and direct fitness benefits. In groups with low relatedness, we expect helpers to be benefiting from direct fitness benefits for the behavior to be stable. One of the primary ways relatedness can accrue in a group is by limited dispersal leading to offspring settling near or with their parents and allowing indirect fitness effects to drive the evolution of cooperation and group formation (Shen et al. 2017; Downing et al. 2020; Kay et al. 2020).

In marine fishes, kin selection was long dismissed as an explanation for the formation of social groups. The rationale for this dismissal was that almost all marine fishes have a larval dispersal phase that should break up parent-offspring and sibling-sibling pairs, preventing mechanisms that rely on relatedness from explaining social groups (unless there are strong kin-recognition mechanisms). Nevertheless, kin selection has come back into focus in studies of marine fish social systems as larval dispersal for some marine fishes is limited and the probability of successful dispersal often decreases sharply with distance from their parental group (D’Aloia et al. 2015; Rueger et al. 2020). For example, in the neon goby *Elacatinus lori*, the probability of successful dispersal declines exponentially as a function of distance and the median dispersal distance is just 1.7 km (D’Aloia et al. 2015). In some species with limited dispersal, a high proportion of larvae either do not leave or return to their natal reefs, ie they “self-recruit” (Jones et al. 1999, 2005). This may lead to relatedness patterns that allow kin selection to play a role in social group formation (D’Aloia and Neubert 2018).

For kin selection to work, relatedness does not need to be high, and no parent-offspring or sibling relationships need to exist in the groups. Instead, more distant relationships will suffice as long as relatedness within the group is higher than between random members of the population (Eberhard 1975; Taylor 1996). In the pajama cardinalfish, *Sphaeramia nematoptera*, relatedness between individuals declines sharply over approximately one kilometer and more offspring than expected by chance recruit to their natal reef (within ≈ 500 m) and close to their parents (within ≈ 1 m; Rueger et al. 2020). In the emerald coral goby, *Paragobiodon xanthosoma*, which lives in groups with strict hierarchies divided into breeders and non-breeders, relatedness within groups confined to coral heads is significantly higher than in the population (Rueger et al. 2021). These examples illustrate that kin selection should be considered as an explanation for social group formation in marine fishes.

The clown anemonefish, *Amphiprion percula*, has emerged as a model to test social evolution hypotheses in marine fishes (Roux et al. 2020; Buston et al. 2022). *Amphiprion percula* live in an obligate mutualism with giant sea anemones, which they use as protection, breeding sites and a nutrient source (Mariscal 1970; Fautin 1992; Verde et al. 2015). Clown anemonefish have a bipartite life cycle with a dispersive larval phase of 10 to 12 d and a mean dispersal distance of 13 to 19 km (Almany et al. 2017). At some sites self recruitment is high, with a third of settled juveniles returning to the same reef structures as their parents (Jones et al. 2005). Once *A. percula* settle into their adult habitats (sea anemone hosts), the habitat is saturated and it is risky for the fish to leave their anemone, which results in there being high ecological constraints in this system. Within each anemone, *A. percula* form groups with a dominant breeding pair and zero to six non-breeding subordinates (Fricke 1979; Mitchell 2003a; Buston 2004a). Non-breeders tolerate their position because they stand to inherit breeding positions in the future (Buston 2004a), and because of the strong social and ecological constraints preventing them from pursuing alternative options (Rueger et al. 2018; Branconi et al. 2020). However, the other side of the coin, why breeders share their territories and resources with subordinate group members is not yet fully understood.

There are few successful attempts to explain why dominant breeding *A. percula* accept subordinate non-breeders in their group. Potential explanations include: H0) the null hypothesis, that dominants neither accrue benefits nor suffer costs from the presence of subordinates (Buston 2004b); H1) the eviction constraints hypothesis, that toleration of subordinates will be favored by selection if it is difficult or dangerous to evict, but dominants can and do evict subordinates under some circumstances (Buston 2003; Rueger et al. 2018; Branconi et al. 2020); H2) that dominants benefit directly through alloparental broodcare, but no alloparental care has been observed in wild anemonefishes (Fricke 1979; Yanagisawa and Ochi 1986; Mitchell 2003b; Buston 2004b; Phillips et al. 2020); H3) the mutualist mediated benefits hypothesis, that subordinates might enhance the growth and survival of the anemone territory (Rueger et al. 2021), though this hypothesis remains untested; H4) dominants benefit in the future through rapid mate-replacement should a breeder die, but the benefits through rapid mate replacement were found to be minimal (Buston 2004b); and H5) dominants receive indirect genetic benefits via their relatives inheriting their territory (Buston 2004b). In a previous study, relatedness within groups was found to be zero based on an analysis of nine groups using seven polymorphic microsatellite markers (Buston et al. 2007). However, like some other coral reef fishes, in *A. percula* the probability of successful dispersal has been shown to decline exponentially as a function of distance (Buston et al. 2011 ; Almany et al. 2017), making elevated relatedness within groups a possibility. We propose that subtle patterns of relatedness may lead to inclusive fitness contributing to why dominant breeders accept subordinates in groups of *A. percula*.

Here, we study relatedness within 203 groups of the clown anemonefish, *A. percula*, using 40 polymorphic microsatellite markers, to test if kin-selection could explain why dominant breeders accept subordinate non-breeders in their groups. Our specific objectives are to 1) test if relatedness is higher within social groups of *A. percula* than in the population as a whole; 2) test if relatedness is predicted by location of the dyad (same group, same reef, different reef) or dyad type (breeder/breeder, non-breeder/breeder, non-breeder/non-breeder).

## Methods

### Sampling

**Fig. 1.**
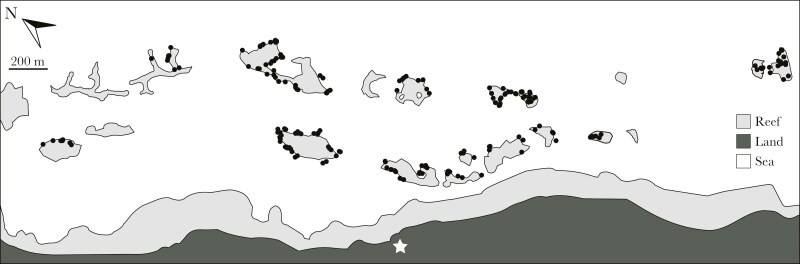
Groups of *Amphiprion percula* on inshore reefs close to Mahonia Na Dari Research and Conservation Centre (star), in Kimbe Bay, Papua New Guinea (Black dots; N = 203). Here, anemones generally occur on the reef slope surrounding pinnacles, with each pinnacle being separated from the next by deeper water that is inhospitable for corals, anemones, and anemonefishes.

In November-December 2019 we sampled 699 *Amphiprion percula* from 203 distinct groups on 15 inshore reefs in Kimbe Bay, Papua New Guinea (Fig. 1). Each group occupied a single magnificent sea anemone, *Radianthusmagnifica,* at 2 to 20 m depth. The number of individual fish per group ranged from 1 to 6, with a mean ± s.e. of 3.45 ± 0.06. Most groups (n = 180 out of 203) included at least one subordinate non-breeder, and only a few groups included just the breeding pair (n = 21) or a single individual (n = 2). All work was conducted with the approval of Boston University Institutional Animal Care and Use Committee (IACUC #201900075).

To take a tissue sample for genetic analyses, individual fish were caught while SCUBA diving using hand nets and transferred underwater to a zip-lock plastic bag. We used sharp scissors to remove a small part (approximately 3 mm x 3 mm) of the soft tissue at the end of the caudal fin. We then transferred the fish back to their host anemone. The fin grows back fully within 2 to 3 wk (pers. obs. Rueger). Fin-clipping can temporarily reduce individual reproductive output (Barbasch et al. 2021)  but does not appear to influence individual survival or long-term population size (Salles et al. 2016).

### Isolation of microsatellite loci

Screening of an *A. percula* assembled genome (Genbank assembly GCA_003047355.2) for microsatellite loci was conducted at Cornell University in 2022. Twenty-four assembled chromosomes were each screened for tetrameric microsatellite repeats (minimum perfect repeat length = 5) and primers were designed with msatcommander (v. 1.08-beta) software. Primer lengths ranged between 18 and 24 bp, primer Tms between 58 °C and 62 °C, and optimal PCR product size was 190 to 220 bp. Sixty microsatellite loci were used for multiplex genotyping, from 14 chromosomes (no more than five loci from any single chromosome).

### Marker development and multiplex PCR

All individuals were sequenced at 60 microsatellite loci (three multiplex mixes, 20 loci per mix) using a multiplex PCR protocol for targeted amplicon sequencing. Multiplex PCR and Nextera barcoding methods were used (see, D’Aloia et al. 2017). We amplified the loci with multiplex PCR reactions using QIAGEN Type-It microsatellite kits and the primers listed in Table S1. Samples were pooled across multiplexes and Illumina’s S5 and N7 Nextera primers were used to run a barcoding PCR. The sequencing library was prepared by pooling all barcoded individuals, which were then size-selected with Ampure XP (Beckman Coulter). Scoring alleles by sequence, instead of size alone, reduces homoplasy since there are alleles at loci that are the same size with unique sequences, and the script can resolve those unique haplotypes (Šarhanová et al. 2018). The library was sequenced on a Illumina NextSeq 500 with paired 150 bp reads (mid-output) at the BioResource Center, Cornell University.

### Data processing

We used python scripts (amplicon.py, amplicon_filter.py, https://bitbucket.org/cornell_bioinformatics/amplicon/src/master/) to call genotypes at each microsatellite locus. Default commands were used except the following: -c 4, -a 0.003, -l 80, and –r 5. A minimum of five reads were required for each allele; otherwise, the diploid genotype was re-coded as missing data. To retain only the highest quality markers and individuals, we first excluded loci missing >20% of individuals, then individuals with >20% missing loci. We used Exact Tests in GenePop to exclude markers out of Hardy-Weinberg-Equilibrium (HWE) (Rousset 2008). After the filters were applied, 683 individuals remained, analyzed at 58 loci. Our final dataset contained 683 individuals and 40 markers in HWE (Suppl. Table S1). Statistical analysis (see below) was conducted using relatedness values based on our final dataset.

### Relatedness

We used the R package *Demerelate* to calculate pairwise relatedness for all pairs of individuals (hereafter called dyads) in the population (Kraemer et al. 2017) . To compare different relatedness estimators, we used the *compareestimators* function in the R package *related* (Csilléry et al. 2006; Pew et al. 2015). This function simulates dyads of known relatedness from the allele frequency in a given population (1000 each of full siblings, half siblings, parent–offspring and unrelated dyads) and compares estimated relatedness with expected relatedness values using Pearson’s correlation coefficient. The estimators tested are the most commonly used in studies of pairwise relatedness; Lynch-Ritland (L-R; Lynch and Ritland 1999), Lynch-Li (L-L; Li et al. 1993), Queller-Goodnight (Q-G; Queller and Goodnight 1989) and Wang (W; Wang 2002). For our marker set and sample population, the Q-G and W estimators performed best (L-R: r = 0.821, L-L: r = 0.864, Q-G: r = 0.871, W: r = 0.870, Figure S1). Because some of our markers were close to biallelic, for which the Q-R estimator is not defined, we decided to use the Wang estimator (Wang 2017). Fourteen pairwise relatedness values out of a total of 234,268 pairwise values were higher than the simulated 99^th^ percentile of values for full siblings (r = 0.73). Because these values did not seem to be part of the same continuous distribution but rather formed a discrete cluster with a mean of 0.96 (± 0.02), we suspect that they originated from field or laboratory errors, rather than biologically meaningful processes. The 14 pairwise samples were associated with 19 groups of *A*. *percula*, which were removed from subsequent analysis, leaving 184 groups and 201, 937 pairwise relatedness estimates.

### Statistical analysis

All statistical analysis was done in R version 4.3.0 (R core Team 2023) .

To study whether pairwise relatedness estimates change with spatial scale and dyad type, we fitted multimembership models using the *lmerMultiMember* package (van Paridon et al. 2025). Dyad relatedness was used as the response variable, relative location of the dyad (same group, same reef but not the same group, not the same reef) and dyad type (breeder/non-breeder, breeder/breeder, non-breeder/non-breeder) were the predictor variables. We also included the interaction between location and dyad type, since investigating the role of kin selection in group formation requires understanding relatedness patterns within groups specifically. To account for the fact that all individual fish are members of multiple dyads, a membership matrix with individual fish ID as column and individual dyad as row was constructed and used as weight matrix. Assumptions were checked using the *model_check* function in the *performance* package (Lüdecke et al. 2021). Conditional and marginal R^2^ were calculated using Nakagawa’s R^2^ in the *performance* package. Statistical significance of individual predictors was assessed with a likelihood ratio test between the model with the focal predictor and a model without the focal predictor.

## Results

In *A. percula*, mean pairwise relatedness (± standard error) within groups was 0.0017 ± 0.0003.

**Fig. 2.**
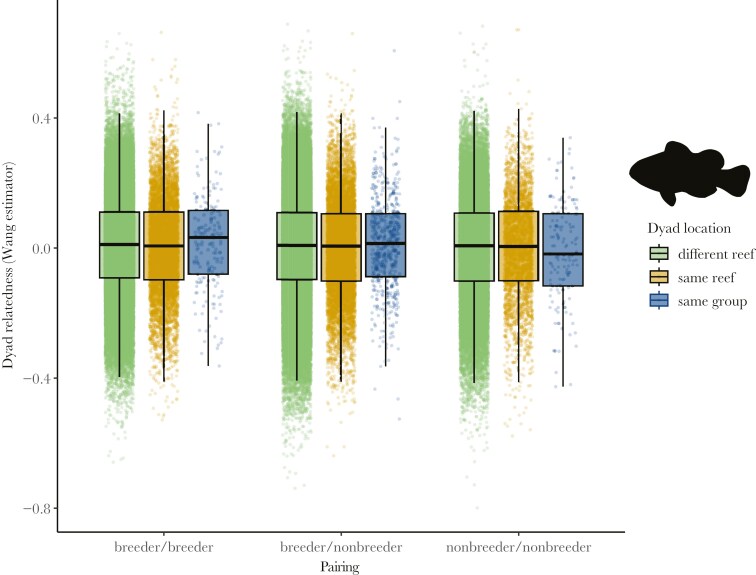
Pairwise relatedness estimates for nine types of dyads of *Amphiprion percula* in Kimbe Bay. Dyads are categorized based on location of individuals within the dyad [different reef = light green (N = 183,224); same reef but different group = orange (N = 17,187); same group = blue (N = 866)] and relative maturity. Central bar represents the median; boxes represent lower and upper quartiles; whiskers represent ± 1.5 interquartile range; raw data is also shown.

Estimated mean (± standard error) relatedness for dyads in the same group was 0.0087 ± 0.0051, for dyads in different groups on the same reef was 0.0025 ± 0.0012, and for dyads on different reefs was 0.0042 ± 0.0004. Relatedness was not predicted by relative dyad location (same group, same reef, different reef) (χ^2^ = 2.318, df = 2, p = 0.314), dyad type (breeder/breeder, breeder/non-breeder, non-breeder/non-breeder) (χ^2^ = 0.178, df = 2, p = 0.915) or the interaction between location and type (χ^2^ = 0.771, df = 4, p = 0.9422) (Fig. 2). The fixed effects explained none of the variance in the data (R_c_ = 0.252, R_m_ = 0.000).

## Discussion

We found no evidence of kin selection playing a structural role in social group formation in *A. percula*. Pairwise relatedness within a group was no higher than pairwise relatedness within a reef or within the population and there was no relationship between pairwise relatedness and dyad types based on either breeding status. On the one hand this result is not surprising because the dogma in marine ecology is that dispersal occurs over 10 to 100 s of kilometers for many fish species (Cowen et al. 2006), and a preliminary assessment based on a limited sample and small microsatellite panel found individuals within *A. percula* groups to be unrelated (Buston et al. 2007). On the other hand, this result is surprising because robust small-scale relatedness patterns were recently found in other coral reef fishes with similar social systems (Rueger et al. 2020; Rueger et al. 2021), and kin selection has repeatedly been shown to be the primary driver of group formation (Downing et al. 2020; Kay et al. 2020). Likely, social group formation has a multifaceted explanation in marine environments, where indirect effects of inclusive fitness may only play a minor role, and the direct fitness effects may be of primary importance. These results do not preclude kin-directed help or kin recognition within *A. percula* groups. However, these effects, if present, do not seem to be playing a major role in their social evolution. Since relatedness is not higher in groups than it is in the population, other explanations need to be considered as to why dominant breeders accept subordinate non-breeders in clown anemonefish social groups.

First, it was plausible that toleration of subordinates might have been favored by selection if it was difficult or dangerous to evict, and if the benefits of toleration outweigh the costs. However, recent experimental studies have confirmed that dominants can and do evict subordinates under some circumstances (Buston 2003): subordinates that attempt to reproduce are more likely to be evicted than those that do not (Rueger et al. 2018); subordinates that attempt to grow large and challenge the breeders are more likely to be evicted than those that do not (Branconi et al. 2020). Similar evictions are seen in cichlid and mongoose groups (Cant et al. 2010; Dey et al. 2015). This suggests that, in clown anemonefish, dominant breeders tolerate subordinate non-breeders either because they remain small, immature, and do not use many resources (null hypothesis), or because they accrue some other benefit from their presence. A second possibility was that breeders might benefit from the presence of non-breeders if they were to provide alloparental care. However, there is no evidence of non-breeders helping breeders in this way, when both members of the breeding pair are present, in any anemonefish studied to date (Fricke 1979; Mitchell 2003b; Buston 2004b)

The third plausible hypothesis for why dominants would tolerate subordinates is that subordinates might enhance the growth and survival of the anemone territory, defending against predators, cleaning, aerating and providing nutrients (Cleveland et al. 2011; Szczebak et al. 2013; Rueger et al. 2022a; Yllan et al. 2024). A large, healthy anemone may facilitate growth of the dominant breeders (Buston 2002; Rueger et al. 2022b), such that the size of the breeders and anemone hosts becomes positively correlated (Fautin 1992; Chausson et al. 2018), which may enhance the reproductive success of the breeders (Barbasch et al. 2020). Evidence from wasp-like queuing system models demonstrates that even small reductions in mortality from improving the habitat can have large fitness benefits to the dominant (Toyoizumi and Field 2014). Subordinate anemonefish may be motivated to engage in behavior that enhances the anemone habitat to avoid eviction. In some cooperative breeders, dominants adjust the level of aggression depending on how much help subordinates provide: cichlid fishes (Taborsky 1985), *Polistes* wasps (Reeve and Gamboa 1987), naked mole rats (Reeve 1992), and fairy wrens (Mulder and Langmore 1993). In anemonefishes, when subordinates are experimentally prevented from helping in this way, they are more likely to receive aggression from dominants, suggesting that some ‘pay-to-stay’ mechanism plays a role in anemonefish group dynamics (Gaffney et al. *in review*). Alternatively, subordinate anemonefish may help enhance the growth and survival of the anemone because they stand to inherit the host as a breeding territory as they move up the hierarchy (Buston 2004a). However, whether cooperative behaviors that benefit the mutualistic host lead to long-term benefits for dominant breeders and whether these mutualist mediated benefits can contribute to an explanation of group formation remains to be tested.

A fourth possibility for why dominants tolerate subordinates is that they may function as rapid mate replacements when breeders die (Fricke 1979). The *mean* time to replace a mate in *A. percula* was not found to be very different when a subordinate was present versus when it was not: females that tolerate a single non-breeder were estimated to have a relative fitness approximately 2% higher than females that do not tolerate non-breeders (Buston 2004b). However, according to within-generation evolutionary bet-hedging, it may be that subordinates significantly reduce the *variance* in mate replacement time (Rubenstein 2011). Since recruitment in the marine environment is largely a stochastic process (Pinsky et al. 2010), the variance of time to mate replacement is high and there is a risk of waiting for a long time for a recruit to arrive. This hypothesis remains to be tested.

In conclusion, kin selection is unlikely to play a role in the social group formation of anemonefishes. Alternative hypotheses need to be rigorously tested using long term observations as well as experimental studies to uncover why dominant breeders accept subordinate non-breeders in this system. These alternatives should also focus on direct fitness benefits that dominant pairs accrue from the presence of the subordinates, and the explanation likely relies on several direct fitness benefits working together. Anemonefishes provide a great opportunity to test less-studied aspects of social group formation, such as evolutionary bet-hedging and pay-to-stay models, without the confounding factor of high within-group relatedness.

## Supplementary Material

araf075_suppl_Supplementary_Material

## Data Availability

Analyses reported in this article can be reproduced using the data provided by Rueger et al. 2025.
