# Supplementary material for: No evidence for kin selection as an explanation for social group formation in clown anemonefish
Source: Behav Ecol. 2025 Jun 29;36(4):araf075. doi: 10.1093/beheco/araf075 (PMC12277695; doi:10.1093/beheco/araf075)
Supplement: araf075_suppl_Supplementary_Material [file araf075_suppl_supplementary_material.docx]

**Supplemental Material**

Marker table

**Table S1.** Polymorphic microsatellite markers developed for *Amphiprion percula*. Primer sequence, repeat motif and repeat count (from the published genome), size range (base pairs excluding forward and reverse primers), number of alleles (Na), observed (Ho) and expected (H_E_) heterozygosities, deviation from the exact test of Hardy–Weinberg-Equilibrium (HWE), and fixation index (F_is_) are presented for each locus. All forward primers were synthesized with 5’ Nextera tag TCGTCGGCAGCGTCAGATGTGTATAAGAGACAG, all reverse primers with tag GTCTCGTGGGCTCGGAGATGTGTATAAGAGACAG (tags not shown in Table S1).

| ATCC | Sequence 5’-3’ | Repeat motif ^count^ | Size range (bp) | N_a_ | H_o_ | H_E_ | HWE | F_is_ |
| --- | --- | --- | --- | --- | --- | --- | --- | --- |
| Aperc_c1_16 | F: ACCAACGCAGAGATACTAAATGACC  R: AGGACAGATGACTCACTCGATAGC | AGAT ^10^ | 143-195 | 6 | 0.575 | 0.573 | 0.131 | -0.003 |
| Aperc_c1_12 | F: CAACTCACCTACCCTCCCTTGAAG  R: CTGTGTGGAGCTAATGTACTGGTG | AAAG ^10^ | 152-180 | 3 | 0.221 | 0.238 | 0.202 | 0.073 |
| Aperc_c2_7 | F: CTGCCACCATGACCCAACACTG  R: AGCATGCACTCCTTTCCAGTAATC | ATCC ^11^ | 170-198 | 6 | 0.466 | 0.476 | 0.672 | 0.022 |
| Aperc_c2_44 | F: GTTTGTGTTGTTACCTCGGGACTG  R: TTACAGCTCGGTCCTCACAAAGAC | ACAG ^11^ | 151-215 | 4 | 0.375 | 0.361 | 0.107 | -0.040 |
| Aperc_c3_7 | F: TCAGCCTGGCCACTACTTAAATAC  R: AATGTGACTAACCTGCCTGTCCTG | ACAG ^10^ | 155-179 | 4 | 0.201 | 0.212 | 0.389 | 0.051 |
| Aperc_c3_34 | F: TCTTGGAGTACTGCTAAGAAAGACAC  R: CAGCTGGACCACAACCCTAATAAG | ATCC ^9^ | 161-177 | 5 | 0.592 | 0.598 | 0.666 | 0.011 |
| Aperc_c3_39 | F: TTGTCTCTGTGTAGTTCTGCGATAG  R: CAATCGGTGCACTTGTATGTCCTG | ATCC ^9^ | 137-161 | 4 | 0.535 | 0.528 | 0.887 | -0.013 |
| Aperc_c4_30 | F: AGCGACGGTTTAGCTCCCATTAAG  R: CCATCTGATACCATCTCCACCGTC | AATG ^9^ | 153-181 | 7 | 0.488 | 0.513 | 0.082 | 0.049 |
| Aperc_c4_24 | F: CAGGAGCAAACAGAAGCAGTGATG  R: TGTCATTATCACCACTGCAGTCTG | AGAT ^9^ | 170-234 | 7 | 0.775 | 0.752 | 0.930 | -0.030 |
| Aperc_c4_27 | F: CTCATGAGCAGTCTTCGTGTTTGC  R: TCTTCACTAGTGGACAGCACAGAG | ATCC ^9^ | 176-184 | 3 | 0.186 | 0.187 | 0.863 | 0.004 |
| Aperc_c4_10 | F: GAAGGCATCAACTTGTTCTAACTGC  R: GCACTAATGAGGTATTGATGAACGGC | ACAG ^10^ | 158-170 | 7 | 0.447 | 0.439 | 0.096 | -0.018 |
| Aperc_c5_15 | F: TGTTGCGAGGTGTTTGTGATACTG  R: GCCCTGCGCTGTCTGTCTG | ACAG ^10^ | 147-191 | 5 | 0.700 | 0.676 | 0.584 | -0.035 |
| Aperc_c5_32 | F: GAGAGATCAAGGCTGGGAGAGG  R: GAGTTTGCCCACCGTTTATTCCTG | AAAG ^9^ | 158-170 | 7 | 0.712 | 0.723 | 0.852 | 0.016 |
| Aperc_c6_29 | F: GCCCACAAACAAGCGGAGG  R: AGTGGATGGACTGGGAAACTAACC | AAAG ^9^ | 152-160 | 3 | 0.502 | 0.494 | 0.854 | -0.016 |
| Aperc_c6_1 | F: TGTTGAAATTGTCCAACACCACCC  R: ACACTACAAAGACACAACCGATGAG | ACAG ^11^ | 129-165 | 7 | 0.680 | 0.680 | 0.581 | 0.000 |
| Aperc_c6_25 | F: AATGGCATCTGTGTGCGTTTGATC  R: TCACCTTTGAGTGTTGTAAAGTGCTC | AATC ^10^ | 136-160 | 7 | 0.584 | 0.607 | 0.555 | 0.038 |
| Aperc_c7_34 | F: TGTCAGGAAGGTAATATGGAGGTGG  R: ACAAGCGTACTGTAGTTGTTGGTC | ATCC ^9^ | 176-212 | 4 | 0.452 | 0.450 | 0.721 | -0.005 |
| Aperc_c7_26 | F: TCCCTGGGACCACATGAATAAATC  R: TCACATCTATCACACCTACATAGCCC | AATG ^10^ | 144-168 | 4 | 0.382 | 0.387 | 0.145 | 0.011 |
| Aperc_c7_3 | F: ATCACACATCACCGCCACTGATTC  R: ATAACACGGAGGCAAACAAGAGTG | ACAG ^11^ | 139-155 | 4 | 0.697 | 0.711 | 0.180 | 0.020 |
| Aperc_c11_44 | F: ACTGTGCTCCATACTGACTTTCCC  R: TCTTTCCTGTCCAAGCATCGTCTC | ATCC ^9^ | 153-217 | 2 | 0.279 | 0.276 | 0.841 | -0.008 |
| Aperc_c11_41 | F: AGCAGAATGGTAGATAACTGAATGGC  R: TAAGATAGGAAGTGGTCAGGCTGC | AGAT ^9^ | 145-157 | 4 | 0.520 | 0.538 | 0.646 | 0.034 |
| Aperc_c11_35 | F: GTAGCGGCTCATCAGGACCATC  R: TGTCTGTCCGTCCTCCTGTC | ACAG ^9^ | 143-163 | 4 | 0.693 | 0.697 | 0.332 | 0.006 |
| Aperc_c11_26 | F: TGAACGAGTTTGTCAAGATGTGGAG  R: CATACTGCTTGCAACAGCCTGTAC | AGAT ^10^ | 171-231 | 4 | 0.606 | 0.567 | 0.120 | -0.069 |
| Aperc_c12_23 | F: GGATGGACAAACAGACATGGACAG  R: GCGGAGTCTTGGATGAGTTATGTG | ACGG ^9^ | 112-168 | 9 | 0.682 | 0.703 | 0.054 | 0.030 |
| Aperc_c12_12 | F: AACCTTTGCACTGTTGAGTCTTGC  R: TCCCAAACCTGCCCATATAACTTC | ATCC ^10^ | 154 | 4 | 0.029 | 0.029 | 1.000 | -0.011 |
| Aperc_c12_30 | F: AGTATCTAGCCAGTCTTTGTCGCC  R: GCCTTATGATAAACTGTGATTGGGTG | ATCC ^9^ | 143-155 | 3 | 0.515 | 0.501 | 0.906 | -0.028 |
| Aperc_c13_18 | F: ACTAACTTACCTGCATCCTCAAATCC  R: TTTCAAACTGGCAGCCTTTCCTAC | AATC ^10^ | 145-165 | 7 | 0.704 | 0.692 | 0.605 | -0.017 |
| Aperc_c13_10 | F: GCCGTGTTGCATAAATTCCTGGAG  R: TGACCTTGCCCAATATGTCGTCTG | AAAC ^11^ | 144-172 | 9 | 0.720 | 0.721 | 0.831 | 0.001 |
| Aperc_c13_16 | F: TGTATGAAATGTGCAAAGCTTCACAC  R: TTAGAGCAGCAGATAAAGAGACTGTC | ATCC ^10^ | 149-161 | 5 | 0.265 | 0.261 | 0.918 | -0.016 |
| Aperc_c14_34 | F: TAGAAACAGGCAGCATGAAGGAGG  R: CTGCTGTCAAACCTTTCACCTTCC | AAAG ^9^ | 141-173 | 4 | 0.526 | 0.519 | 0.981 | -0.013 |
| Aperc_c14_12 | F: TGTGACAGACTGGCGACCTG  R: TGGTTCTGATATTGCCATACCCTTTG | ATCC ^6^ | 152-171 | 7 | 0.679 | 0.680 | 0.072 | 0.001 |
| Aperc_c15_17 | F: GCTGGAGATACAAGGTTTCCACAC  R: GAGACGACGGACCGGAAACC | AATC ^10^ | 157-165 | 2 | 0.007 | 0.007 | 0.924 | -0.004 |
| Aperc_c15_31 | F: TTCAGGCCTGGTCCATGTTACATC  R: ATGTGAGCAGTTGTCCGAGAATATAC | ACTG ^9^ | 116-149 | 9 | 0.635 | 0.612 | 0.157 | -0.039 |
| Aperc_c15_8 | F: ATGTGGAAGTGACTGGATACCTTG  R: GAAGTGACACCAACTACATCGTGC | ATCC ^11^ | 162-188 | 5 | 0.261 | 0.248 | 0.684 | -0.052 |
| Aperc_c15_15 | F: TCATTTGGTTGTGACATGCAGAGG  R: GCGCTTCTAAGATATTTCCCAGATGG | ATCC ^10^ | 146-166 | 5 | 0.692 | 0.707 | 0.115 | 0.021 |
| Aperc_c16_11 | F: CAGCCACATGTTTGACACCCTTG  R: CTGCTTTGACTAGATGGGTTGAGG | ACAG ^10^ | 151-179 | 5 | 0.196 | 0.186 | 0.681 | -0.056 |
| Aperc_c17_35 | F: GCAGTGAGCGTTACAAATGTAGC  R: CGCCCACCTTTCCATCTGTCC | ATCC ^9^ | 148-172 | 3 | 0.490 | 0.478 | 0.770 | -0.027 |
| Aperc_c17_28 | F: GATTCTTACTGTAGTGGAGCTGAGAG  R: CCACTTTAAGCCTTGGCTTGAGAC | ACAT ^9^ | 165-197 | 6 | 0.414 | 0.416 | 0.911 | 0.004 |
| Aperc_c17_8 | F: GGTCAATGTGATCTTGGAACGTGC  R: CACTCACTTTGGGTTCGCATCC | ACAG ^10^ | 137-149 | 3 | 0.132 | 0.136 | 0.371 | 0.029 |
| Aperc_c17_39 | F: TCAGGTGAATTTAGATGGAGCAGTG  R: GATGGACGGACAGATGATGGAC | ATCC ^9^ | 127-157 | 4 | 0.236 | 0.242 | 0.898 | 0.025 |

Estimator simulation


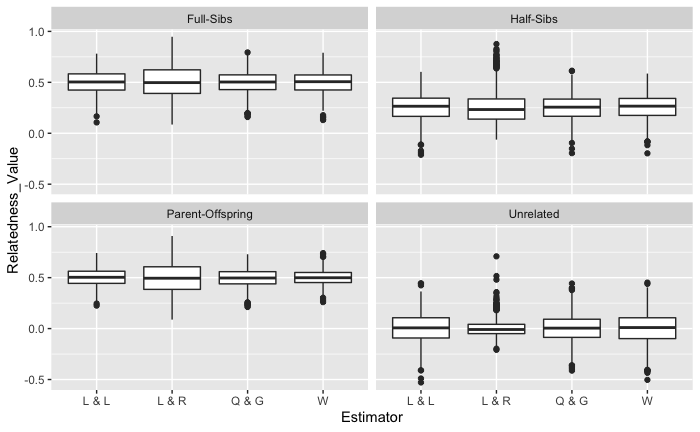


**Figure S1**. Simulation to compare relatedness estimators based on 1000 dyads of full-sibs, half-sibs, parent-offspring and unrelated individuals. The estimators tested were Lynch-Ritland (L-R), Lynch-Li (L-L), Queller-Goodnight (Q-G) and Wang (W).
